# A polycystic variant of a primary intracranial leptomeningeal astrocytoma: case report and literature review

**DOI:** 10.1186/1477-7819-5-72

**Published:** 2007-06-23

**Authors:** Antonio De Tommasi, Giuseppe Occhiogrosso, Claudio De Tommasi, Sabino Luzzi, Antonella Cimmino, Pasqualino Ciappetta

**Affiliations:** 1 Department of Neurology and Neuropsychiatry Sciences, Chair of Neurosurgery, University of Bari, 70124 Bari, Italy; 2 Department of Anatomical and Cellular Pathology, Chair of Pathology, University of Bari, 70124 Bari, Italy

## Abstract

**Background:**

Primary leptomeningeal astrocytomas are rare intracranial tumors. These tumors are believed to originate from cellular nests which migrate by means of aberration, ultimately settling in the leptomeningeal structure. They may occur in both solitary and diffuse forms. The literature reports only fifteen cases of solitary primary intracranial leptomeningeal astrocytomas.

**Case presentation:**

The authors report the case of a seventy-eight year-old woman with a polycystic variant of a solitary primary intracranial leptomeningeal astrocytoma. The first neurological signs were seizures and aphasia. CT and MRI scans demonstrated a fronto-parietal polycystic tumor adherent to the sub arachnoid space. A left fronto-temporo-parietal craniotomy revealed a tight coalescence between the tumor and the arachnoid layer which appeared to wrap the mass entirely. Removal of the deeper solid part of the tumor resulted difficult due to the presence of both a high vascularity and a tight adherence between the tumor and the ventricular wall.

**Conclusion:**

A new case of a solitary primitive intracranial leptomeningeal astrocytoma of a rare polycystic variant is reported. Clinical, surgical, pathologic and therapeutic aspects of this tumor are discussed.

## Background

Moore was the first in 1954 to describe a diffuse form of a primary leptomeningeal astrocytoma (PLA) [1]. These tumors are rare and, in most cases, are associated with a poor survival.

Gliomas very rarely arise from the meninges and, to date, their histogenesis remains controversial. The diffuse forms normally affect the entire leptomeninges, giving a gliomatosis appearance and a macroscopic similarity to meningitis [2], while the solitary forms usually appear as non-glial tumors involving a limited area of the central nervous system [2,3]. These astrocytomas are defined as "primary leptomeningeal astrocytomas" to differentiate them from "secondary leptomeningeal astrocytomas" (SLAs) which represent a secondary leptomeningeal spread from a primitive intra-axial astrocytoma. Hence, the diagnosis of a PLA is mainly based on the radiological exclusion of an astrocytoma elsewhere in the central nervous system. Only fifteen cases of solitary PLAs have been previously reported [4-16] (Table 1). This study reports the case of a solitary PLA localized in the left fronto-parietal area.

**Table 1:** Details of the previous cases reported in literature

**Author & Year**	**Age (yrs), Sex**	**Presentation**	**Histological Findings**	**Site**	**Size (cm)**	**Outcome**
Abbott, 1955 [5]	43, F	headaches, seizures, hemiparesis	astrocytoma	rt hemisphere	NA	well post-op.
Sumi, 1968 [6]	61, M	confusion	astrocytoma	insula	NA	died, 6 mos
Horoupian, 1979 [7]	49, F	seizures, headaches, hemiplegia	astrocytoma	rt fronto-parietal	5	well, 12 mos
Shuangshoti, 1984 [8]	49, F	visual loss	mixed glioma	para-sellar	3.5	no recurrence
Bailey, 1985 [4]	39, M	memory loss, seizure	glioblastoma	lt fronto-parietal	7.5	died, 13 mos
Sceats, 1986 [3]	53, F	ataxia, 8th nerve palsy	astrocytoma	lt cerebello-pontine angle;	2	well post-op.
Kakita, 1992 [9]	74, F	difficulty in walking, sleep	glioblastoma	lt parietal	NA	died, 2 mos
Krief, 1994 [10]	26, F	seizures	glioma	lt parietal	NA	NA
Opeskin, 1994 [11]	59, M	headaches	astrocytoma	cerebellum	2	died, 7 mos
Ng, 1998 [12]	79, M	partial seizures	fibrillary astrocytoma	lt temporo-parietal	5	died, 1 mos
Sell, 2000 [13]	62, M	headache, diplopia, nausea, ataxia	high-grade astrocytoma	rt hemisphere, lt occipital lobe, both thalami	2,5	died, 4 mos
Cirak, 2000 [14]	2, F	weight loss, apnea attacks	astrocytoma	brainstem	NA	NA
Wakabayashi, 2002 [15]	33, M	seizures, headaches	glioblastoma	frontal	6	metastasis to the femur 39 months after craniotomy
	72, M	seizures, headaches	oligodendroglioma	frontal	4	recurrence, 8 mos
	72, F	seizures	glioblastoma	lt parietal	5	recurrence, 11 mos
De Tommasi, 2007 [Present case]	78, F	seizures and aphasia	polycystic astrocytoma	lt fronto-parietal	7	no recurrence, 24 mos

## Case presentation

A seventy-eight year-old woman complained of a two-week history of partial seizures in right arm with diffusion to the entire right side. The seizures had been preceded by a right-sided dysesthesia and dysphasia. At admission, the patient was in G.C.S. 14. Speech ability and cranial nerve functions were normal. No gait disturbances were present. Limb strength and sensory examination were normal. Deep and superficial reflexes were bilaterally normal and symmetrical.

**Figure 1:**
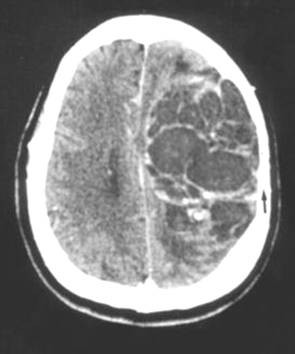
CT scan showing a roundish, hypodense left fronto-parietal mass with a thinness satellite parietal bone (arrow). A contrast enhanced CT scan reveals any septa inside the tumour which give it a polycystic aspect.

A brain CT scan revealed a large, round mass (seven cm. in diameter) in the left fronto-parietal area causing a shift of the midline structures. A thickening of the meninges was detected around the polycystic mass. The left parietal inner bone appeared thin. (Figure 1).

**Figure 2:**
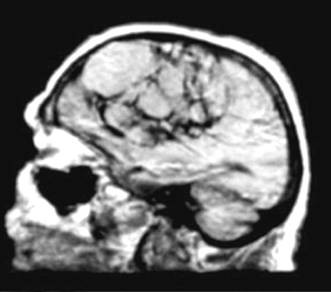
Sagittal T1 weighed MR showing the polycystic fronto-parietal tumour.

A brain MR highlighted the polycystic shape of this mass (Figure 2).

Cerebrospinal fluid (CSF) was studied by means of an intracranial drainage for ICP measurement. Analysis of CSF showed an increased protein level and a reduced glucose level. Cytological examination showed no malignant cells. Gram's and Ziehl-Neelsen stains were negative.

After implantation of a cardiac pace-maker necessary because of hypokinetic arrhythmia, a left fronto-temporo-parietal craniotomy was performed. The meninges adhered tightly to a grey, extra-axial, polycystic neo-formation. Once the meninges were opened, it was possible to penetrate into a large cystic cavity composed of smaller cysts ranging in size between one and three centimetres. A few cysts were communicating whereas others were isolated. The cysts contained a dense yellowish liquid. Furthermore, the cyst walls were quite thin and their inner wall showed a dense, veil-like, white tissue connected with a solid part of the tumor. The core of the tumor consisted almost entirely of a dense, intra-cystic liquid which was suctioned. Removal of the deeper solid part of the tumor resulted difficult owing to the presence of both a high vascularity and a tight adherence between the tumor and the ventricular wall.

The immediate postoperative course was good even though, on the fourth day after surgery, despite carbamazepine, the patient suffered a new episode of dysphasia which spontaneously resolved.

**Figure 3:**
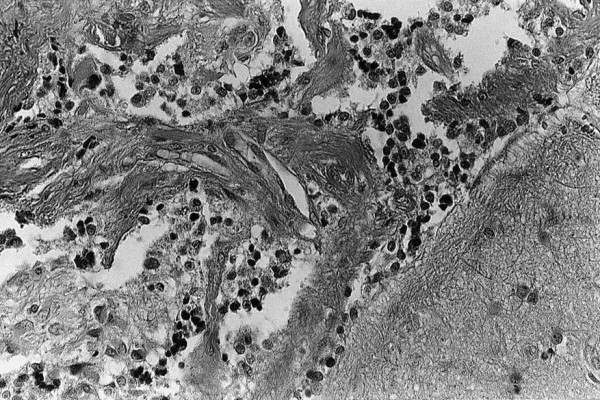
Microphotograph showing the diffuse and fairly dense cellular proliferation as well as the infiltration of subarachnoid space (E&E ×160).

**Figure 4:**
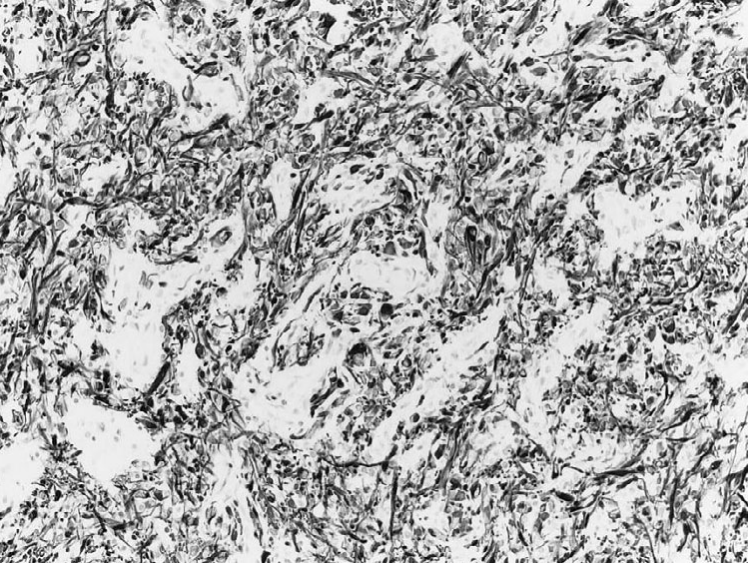
Intense immunohistochemical positivity to glial fibrillary acidic protein (GFAP) confirmed the astrocytic phenotype of the tumour.

Anatomical specimens appeared rust-coloured and were composed of many cellular areas. Microscopic examination revealed leptomeningeal thickening with a diffused, well-differentiated and fairly glial cellular proliferation in the subarachnoid space (Figure 3). Nuclear atypia and hemosiderosis are evident. Intense immunohistochemical positivity to glial fibrillary acidic protein (GFAP) confirmed the astrocytic phenotype of the tumor (Figure 4).

A primary leptomeningeal astrocytoma (W.H.O. grade II) was diagnosed.

The patient was discharged from hospital ten days after surgery in good clinical conditions. At the two-year follow-up, the neurological exam was normal and a total body CT scan revealed no distal metastases.

## Discussion

PLA is considered a neoplastic transformation of glial heterotopias [3,4] represented by nests of glial tissue which, during embryogenesis, separate from the bulk of the central nervous system and remain trapped in the leptomeningeal sheets following an aberrant migration [8,17].

The cellular nests may contain oligodendrocytes, ependymal cells [2,18], as well as neurons, neuroblasts and choroid plexus [17-21]. The different cellular combinations can explain the existence of mixed forms of PLAs, such as a combined form of astrocytoma and ependymoma [20] or astrocytoma, ependimoma and oligodendroglioma [8].

SLAs are believed to have a non-heterotopic origin and are considered a leptomeningeal spread of intra-axial gliomas. These forms occur in 20–25% of patients suffering from extensive cerebral gliomas [22].

Cooper and Kernohan first dictated the main criteria on which to base the diagnosis of PLA: no apparent attachment of extramedullary meningeal tumor to the neural tissue, no evidence of primary neoplasia within the neuraxis and the existence of distinct leptomeningeal encapsulation around the tumor [23].

It is noteworthy that PLAs may have two well established anatomoclinical forms: nodular form, first described by Bailey and Dietrich as "a solitary or focal leptomeningeal gliomatosis, defined by limited tumor masses in cranial or spinal leptomeninges," [4,24] and a diffuse form, first reported by Korein as "an extension, outside the nervous parenchyma, of glial tumor cells over a wide area of the CNS" [25].

The literature reports only fifteen cases of intracranial solitary PLAs which are summarized in Table 1. Seven patients were males and eight females. The mean age of the patients was fifty-two years. Twelve cases were supratentorial and only three infra-tentorial.

Clinical presentation of PLAs includes a variety of neurological symptoms but headache, seizures and increased intracranial pressure are the most common observed clinical signs. In many cases the MRI and CSF studies are unable to clarify the diagnosis of these tumors. Tumor biopsy often becomes imperative to confirm the diagnosis.

The lack of symptom specificity as well as the variability of the clinical onset makes the diagnosis of these tumors difficult. This is why most of the described cases were diagnosed post-mortem.

The reported case represents a new case of solitary intracranial SLA and, in particular, an uncommon polycystic variant of it. The main symptoms were seizure and aphasia. The CT scan and MR scan supported the diagnosis of an extra-axial tumor. The microsurgical dissection demonstrated a strong coalescence between the tumor and the arachnoid layers, which enveloped the polycystic mass entirely. The histological studies confirmed a strong coalescence between the tumor-cell proliferation and the arachnoid layers.

Due to the aforementioned lack of specificity concerning clinical, radiological and CSF analysis, the pre-mortem diagnosis of PLAs still remains a challenge for clinicians, who often have difficulty in differentiating these lesions from infectious or autoimmune pathologies. The literature reports almost 30% of cases with no clinical established diagnosis.

Among infections, chronic tuberculous meningitis is the most difficult differential diagnosis from leptomeningeal tumors. Indeed, even in tuberculous meningitis the clinical onset consists of subacute headache, drowsiness, confusion and meningism. CSF examination typically shows increased proteins, decreased glucose concentration and pleocytosis [26].

Unfortunately, when CSF stains for tuberculous are negative, differential diagnosis between tuberculous or malignant meningitis and leptomeningeal tumors is based solely on the responsiveness to antituberculous chemotherapy. Indeed, these patients are treated with steroids and anti-tuberculous therapy until clinical and radiological improvement has been achieved. The authors are in agreement with Rogers and Wechsler who believe that a search for malignancy is mandatory in those patients who had failed to respond to the anti-infectious and anti-inflammatory treatment although iterative CSF examination has demonstrated no neoplastic cells [27,28].

Other intracranial glial tumors may have a cystic pattern. Among these, pleomorphic xanthoastrocytoma (PXA) is one of the most frequently observed in supratentorial spaces, especially in elderly patients. Indeed, in a series of 20 patients reported by Rostomily all the tumors were supratentorial and 5 were cystic [29]. In a review on 71 cases, Giannini observed that PXAs occur primarily in young patients with a mean age of 26 ± 16 years [30], whereas the mean age of patients diagnosed with a PLA was of 33.79 ± 17.96 years in a series of 29 cases reported by Debono [31].

From a macroscopic point of view, although PXAs are usually superficial in location, they are an intra-axial neoplasm only partly occupying the leptomeninges and with a natural tendency to involve perivascular Virchow-Robin spaces. On the contrary, the diagnosis of a PLA requires "no evidence of primary neoplasia within the neuraxis and the existence of distinct leptomeningeal encapsulation around the tumor" [23].

Since the first description by Kepes in 1979, the diagnosis of PXAs should comply with a few criteria such as: 1) highly pleomorphic tumor cells, 2) presence of lipid-laden, foamy, or "xanthomatous" tumor cells, 3) absence of necrosis, 4) rare or no mitoses, 5) GFAP positivity in some tumor cells, and 6) rich pericellular or stromal reticulin staining [32]. Even though PLAs share an intense positive immunoreactivity to GFAP due to their common glial nature, these tumors show neither "xanthomatous" cells nor that particular meshwork of reticulin surrounding individual or clusters of tumor cells which is characteristic of xanthoastrocytomas, especially in their superficial and leptomeningeal portions [32].

From a therapeutic point of the view, the literature debates chemo and/or radiotherapy protocols for PLA.

To the authors' knowledge, only six cases of these rare tumors were treated with adjuvant chemo-radiotherapy [27,33-37]. Among these, craniospinal radiotherapy was the choice treatment in 2 cases, radiotherapy and chemotherapy in 3 cases and intrathecal chemotherapy alone in the last case. Beauchesne, in treating a seventeen-year-old male patient diagnosed with a diffuse form of PLA, reported a complete clinical remission achieved over fifteen months thanks to cortico-spinal radiation and three courses of an eight-drug systemic chemotherapy (French Society of Pediatric Oncology 91 protocol) [37].

In the reported case the authors decided against adjuvant therapies after radical surgery considering the lack of tested protocols of chemo-radiotherapy in PLAs.

Conclusions

The present case is an interesting new case of a PLA considering both the patient's good outcome observed at the two-year follow-up as well as the fact that no chemo and/or radiotherapy was undergone. The patient's positive outcome suggests that polycystic variants of a PLA might be associated with a better prognosis compared to other PLAs.

## Competing interests

The author(s) declare that they have no competing interests.

## Authors' contributions

ADT: conceived the case report, participated in the surgery as first operator and drafted the manuscript. 

GO: participated in the surgery and in clinical management of the patient.    

CDT: participated in the clinical management of the patient and in the literature review.

SL: participated in the clinical management of the patient and in the literature review.

AC: examined the histological specimens and participated in the literature review.

PC: proofread the manuscript.

All the authors have read and approved the final manuscript.
